# Assessment of serum phenylalanine and tyrosine isomers in patients with ST‐segment elevation vs non‐ST‐segment elevation myocardial infarction

**DOI:** 10.1002/jcla.23613

**Published:** 2020-10-11

**Authors:** Ied Al‐Sadoon, István Wittmann, Szilard Kun, Mercédesz Ahmann, Attila Konyi, Zsófia Verzár

**Affiliations:** ^1^ Doctoral School of Health Sciences Faculty of Health Science University of Pécs Pécs Hungary; ^2^ Department of Medicine and Nephrological Center Medical School University of Pécs Pécs Hungary; ^3^ Heart Institute Medical School University of Pécs Pécs Hungary

**Keywords:** meta‐tyrosine, myocardial infarction, ortho‐tyrosine, oxidative stress, para‐tyrosine, phenylalanine

## Abstract

**Background:**

Under conditions of oxidative stress, hydroxyl radicals can oxidize phenylalanine (Phe) into various tyrosine (Tyr) isomers (meta‐, ortho‐, and para‐tyrosine; m‐, o‐, and p‐Tyr), depending on the location of the hydroxyl group on the oxidized benzyl ring. This study aimed to compare patients with ST‐segment elevation myocardial infarction (STEMI) and non‐STEMI (NSTEMI) and the serum levels of Phe and Tyr isomers at the aortic root and distal to the culprit lesion in both groups.

**Methods:**

Forty‐four patients participated in the study: 23 with STEMI and 21 with NSTEMI. Arterial blood samples were taken from the aortic root through a guiding catheter and from the culprit vessel segment distal from the primary lesion with an aspiration catheter, during the percutaneous coronary intervention. Serum levels of Phe, p‐Tyr, m‐Tyr, and o‐Tyr were determined using reverse‐phase high‐performance liquid chromatography.

**Results:**

Serum levels of Phe were significantly higher distal to the culprit lesion compared to the aortic root in patients with STEMI. Serum p‐Tyr/Phe and m‐Tyr/Phe concentration ratios were both lower distal to the culprit lesion than at the aortic root in patients with STEMI. There were no statistically significant differences with respect to changes in serum Phe and Tyr isomers distal to the culprit lesion compared to the aortic root in patients with NSTEMI.

**Conclusion:**

Our data suggest that changes in serum levels of different Tyr isomers can mediate the effects of oxidative stress during myocardial infarction.

## Graphical abstract

Arterial blood samples were taken from the aortic root and from the culprit vessel segment distal from the primary lesion with an aspiration catheter, during percutaneous coronary intervention (PCI). Serum levels of phenylalanine (Phe), para‐tyrosine, meta‐tyrosine, and ortho‐tyrosine were determined using reversed phase‐ high‐performance liquid chromatography (r‐HPLC). Serum levels of Phe were higher distal to the culprit lesion compared to the aortic root in both group of patients, with statistical significance in patients with ST‐segment elevation myocardial infarction.

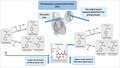

## 1 INTRODUCTION

Acute coronary syndrome (ACS) describes a broad spectrum of clinical symptoms compatible with acute myocardial ischemia, from unstable angina to non‐ST‐segment elevation myocardial infarction (NSTEMI) and ST‐segment elevation myocardial infarction (STEMI).^1^, ^2^ These symptoms are consequences of partial or complete thrombus formation related to the rupture of coronary atherosclerotic plaques.

Reactive oxygen species (ROS) play a vital role in vascular inflammation during atherogenesis, from the onset of fatty streak development to plaque rupture.^3^ ROS or free radicals can be any chemical species (atom, ion, or molecule) that contains a single, unpaired electron in its outer orbit conferring very high reactivity; examples include hydrogen peroxide (H_2_O_2_), singlet oxygen (^1^O_2_), superoxide radical (O·^−2^), and hydroxyl radical (^·^OH).^4^, ^5^, ^6^, ^7^, ^8^, ^9^, ^10^, ^11^, ^12^

**FIGURE 1:**
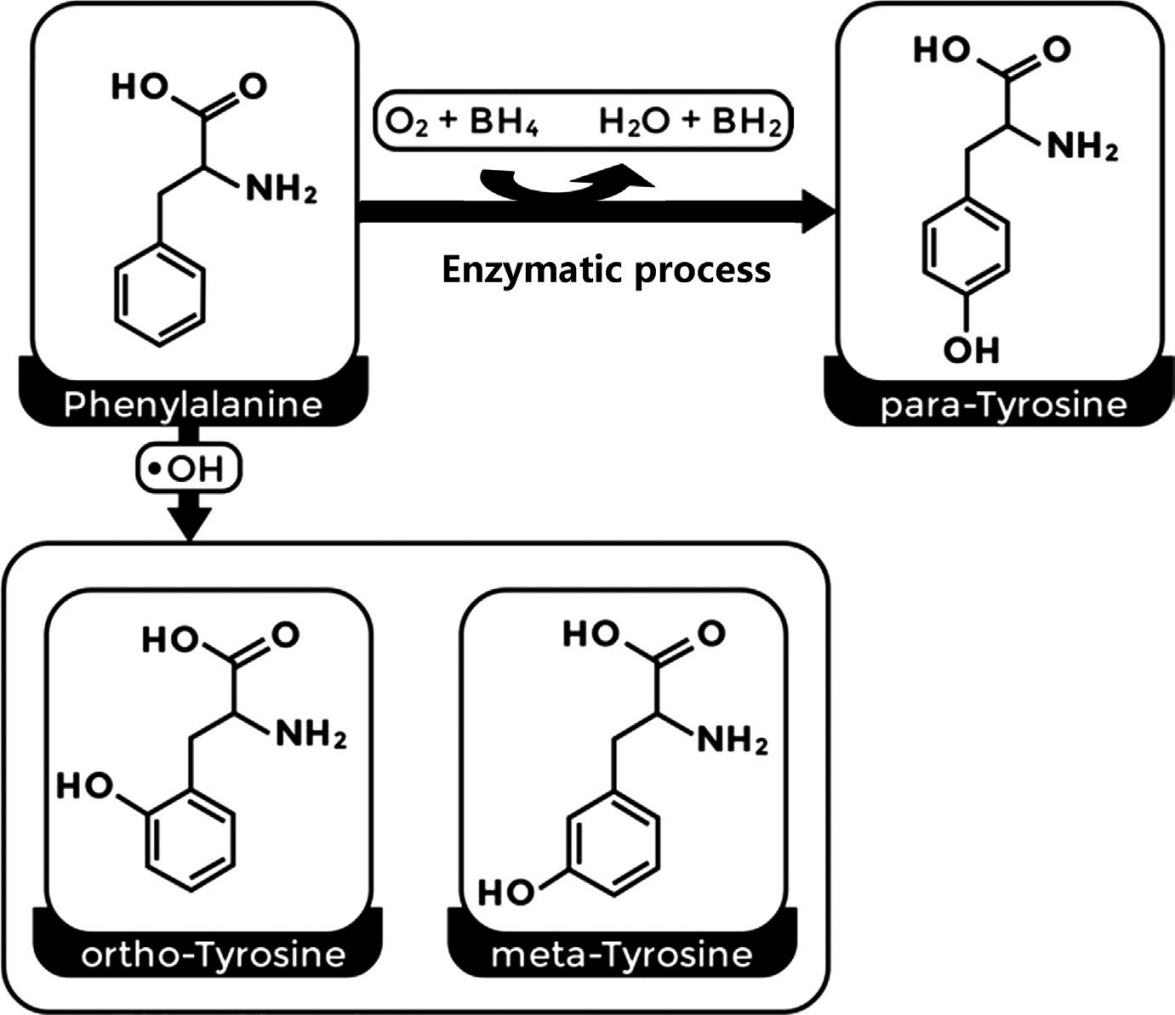
Oxidation of phenylalanine into different tyrosine isoforms due to the activity of the phenylalanine hydroxylase enzyme or under conditions of oxidative stress (mainly via hydroxyl free radicals)

Oxidative stress refers to conditions caused by an imbalance between ROS and antioxidant systems, in which either excessive amounts of free radicals are produced or the antioxidant capacity is decreased; such conditions can result in the oxidation of proteins, lipids, carbohydrates, and DNA.^4^, ^5^, ^6^, ^7^, ^8^, ^10^ Under conditions of oxidative stress in which the levels of free radicals are elevated, hydroxyl radicals can oxidize the benzyl ring of phenylalanine (Phe), producing various tyrosine (Tyr) isomers (meta‐tyrosine, ortho‐tyrosine, and para‐tyrosine; m‐, o‐, and p‐Tyr). These Tyr isomers differ, depending on the location of the hydroxyl group on the benzyl ring (Figure 1).^13^, ^14^, ^15^, ^16^, ^17^, ^18^

According to previous studies,^13^, ^14^, ^15^, ^16^, ^17^, ^18^ Phe can be enzymatically transformed into physiological p‐Tyr. Additionally, p‐Tyr can also be nonenzymatically produced by reactions involving hydroxyl radicals during oxidative stress. However, the enzymatically produced p‐Tyr is more plentiful than the free radical‐derived p‐Tyr. Therefore, p‐Tyr is viewed as the physiological isoform.^13^, ^14^, ^15^, ^16^, ^17^, ^18^ Furthermore, in humans, m‐ and o‐Tyr amino acids cannot be formed enzymatically; instead, they are produced as a result of the reaction between the hydroxyl free radical and the benzyl ring of Phe. Therefore, m‐Tyr and o‐Tyr are viewed as free radical markers,^13^, ^14^, ^15^, ^16^, ^17^, ^18^ which may play a role in chronic inflammation during the initiation and progression of ACS.^16^, ^17^, ^19^ This study aimed to compare patients with STEMI and NSTEMI, and the serum levels of Phe and Tyr isomers at the aortic root and distal to the culprit lesion in both patient groups.

## 2 MATERIALS AND METHODS

### 2.1 Study population

This prospective study was performed according to regulations issued by the local ethics committee (4511/2016) of the Medical Faculty and Doctoral School of the Health Sciences of the University of Pécs and compiled in accordance with the ethical guidelines of the 2003 Declaration of Helsinki. Written consent was obtained from all patients or their nearest relatives after they were informed clearly about the details of the study. The study was conducted on 44 patients diagnosed with ACS who were admitted to the cardiac catheterization laboratory (Department of Interventional Cardiology, University of Pécs Clinical Centre [Pécsi Tudományegyetem/Heart Institute]) between January 1, 2017, and March 3, 2017. Patients with ACS were divided into two groups according to ST‐segment deviation: STEMI and NSTEMI, based on European Society of Cardiology and American College of Cardiology/American Heart Association (ACC/AHA) guidelines.^20^, ^21^, ^22^

The extent of CAD was ascertained by coronary angiography. The extent of CAD was determined according to the number of coronary arteries with obstructive CAD (defined as ≥50% angiographic stenosis): 0‐, 1‐, 2‐, or 3‐vessel disease. On angiography, of course an occlusive lesion easily recognizable as infarct‐related artery. Occasionally, in cases of NSTEMI, to define the culprit lesion is not so easy in patient with multivessel coronary artery disease. Thus, the identification of the culprit lesion is usually achieved by a combination of factors, including angiographies characteristics and information from non‐invasive examinations (ECG, echocardiography). In case of non‐occlusive MI, the ESC/AHA definition was used the determine existing of CAD.

### 2.2 Clinical and biochemical parameters

Personal and medical histories of all study patients were recorded. Arterial blood samples were taken from the aortic root using a guiding catheter and from the culprit vessel segment distal from the primary lesion using an aspiration catheter, during the percutaneous coronary intervention. The database has been published previously.^23^ Serum was obtained by centrifugation (1008 *g*, 10 minutes) and stored at −80°C until further examination. Serum m‐Tyr, o‐Tyr, p‐Tyr, and Phe levels were determined by reverse‐phase high‐performance liquid chromatography (Shimadzu USA Manufacturing Inc) using a C18 silica column (250 × 4 mm) with fluorescence detection (Tyr [*λ*_em_ = 275 nm/*λ*_em_ = 305 nm] and Phe [*λ*_ex_ = 258 nm/*λ*_ex_ = 288 nm]), as described previously.^24^

### 2.3 Statistical analyses

SPSS software, version 22.0 (IBM Corporation), was used for statistical analyses. Continuous variables are expressed as the mean ± standard deviation or median and interquartile range (25%‐75%). Categorical variables are expressed as percentages and frequencies. Differences between the STEMI and NSTEMI groups were assessed using the chi‐square test for categorical variables, Student's *t* test for normally distributed continuous variables, and the Mann‐Whitney *U* test for skewed continuous variables. For pairwise comparisons of each group, the Wilcoxon test was used, depending on the normal distribution. To assess the correlation between the amino acid parameters and baseline characteristics of patients with ACS, we used Spearman's rho test. *P* < .05 were considered statistically significant.

## 3 RESULTS

### 3.1 Baseline characteristics of patients with ACS

Demographics and patient data are summarized in Table 1. Forty‐four patients were included in the study: 23 with STEMI and 21 with NSTEMI. The mean age of the participants was 68.1 ± 9.4 years, and most were female (75.0%). A previous history of hypertension, smoking, and diabetes mellitus was found in 79.5%, 38.6%, and 36.4% of the patients, respectively. Moreover, most patients (84.1%) had one‐vessel disease. There was no significant difference in smoking, diabetes mellitus, and extent of CAD, with the exception of hypertension, between patients with STEMI and NSTEMI.

**TABLE 1:** Baseline characteristics of the study patients

Characteristics	STEMI (n = 23)	NSTEMI (n = 21)	Total (n = 44)	*P* value
Age, y (mean ± SD)	66.87 ± 8.745	69.57 ± 10.201	68.16 ± 9.455	.350
Male/Female, n (%)	6/17 (26.1/ 73.9)	5/16 (23.8/ 76.2)	11/33 (25.0/ 75.0)	.570
Smoking, n (%)	6 (26.1)	11 (52.4)	17 (38.6)	.069
Hypertension, n (%)	15 (65.2)	20 (95.2)	35 (79.5)	.016
Diabetes mellitus, n (%)	7 (30.4)	9 (42.9)	16 (36.4)	.294
Culprit lesions				
LM	1 (4.3)	0 (0.0)	1 (2.3)	.337
LAD	12 (52.1)	1 (4.8)	13 (29.5)	.291
Cx	5 (21.7)	16 (76.1)	21 (47.7)	.255
RCA	9 (39.1)	0 (0.0)	9 (20.5)	.776
Extent of CAD				
One vessel disease	21 (91.3)	16 (76.2)	37 (84.1)	.324
Two vessel disease	2 (8.7)	4 (8.7)	6 (13.6)	
Three vessel disease	0 (0.0)	1 (4.8)	1 (2.3)	

Note

Data are expressed as mean ± SD for the continuous variable and percentages (%) and frequencies (n).

Abbreviations: CAD, coronary artery disease; Cx, circumflex artery; LAD, left anterior descending; LM, left main; NSTEMI, non ST‐segment elevation myocardial infarction; RCA, right coronary artery; STEMI, ST‐segment elevation myocardial infarction.

### 3.2 Comparison of the amino acid parameters for patients with ACS

Serum Phe levels were significantly higher distal to the culprit lesion than at the aortic root (44.7 µmol/L vs 35.5 µmol/L, *P* = .002) in patients with STEMI. Serum p‐Tyr/Phe and m‐Tyr/Phe ratios were significantly lower distal to the culprit lesion than at the aortic root (0.7 vs 0.9 µmol/µmol, *P* = .024; 0.1 vs 0.4 nmol/µmol, *P* = .018, respectively) in patients with STEMI (Table 2).

**TABLE 2:** Serum levels of phenylalanine and tyrosine isomers in patients with STEMI

Parameters	Aortic root	The culprit lesion	*P* value
Serum Phe (μmol/L)	35.5 (26.7‐44.9)	44.7 (39.0‐58.6)	.002
Serum p‐Tyr (μmol/L)	31.2 (26.7‐41.6)	32.0 (30.2‐37.0)	.316
Serum m‐Tyr (nmol/L)	17.6 (10.1‐36.2)	10.39 (7.1‐37.1)	.248
Serum o‐Tyr (nmol/L)	16.6 (6.6‐32.6)	11.9 (6.9‐36.7)	.927
Serum p‐Tyr/Phe (μmol/μmol)	0.9 (0.7‐1.1)	0.7 (0.6‐0.8)	.024
Serum m‐Tyr/Phe (nmol/μmol)	0.4 (0.2‐0.6)	0.1 (0.1‐0.4)	.018

Abbreviations: m‐Tyr, meta‐tyrosine; o‐Tyr, ortho‐tyrosine. All data are expressed as median (IQR: 25‐75%); Phe, phenylalanine; p‐Tyr, para‐tyrosine; STEMI, ST‐segment elevation myocardial infarction.

There were no statistically significant differences with respect to changes in serum levels of Phe and Tyr isomers distal to the culprit lesion compared to the aortic root in patients with NSTEMI (Table 3).

**TABLE 3:** Serum levels of phenylalanine and tyrosine isomers in patients with NSTEMI

Parameters	Aortic root	The culprit lesion	*P* value
Serum Phe (μmol/L)	37.4 (34.0‐46.9)	40.2 (33.6‐47.8)	.768
Serum p‐Tyr (μmol/L)	35.6 (30.5‐40.6)	32.8 (26.5‐40.2)	.205
Serum m‐Tyr (nmol/L)	11.6 (6.7‐57.2)	18.4 (6.1‐38.8)	.498
Serum o‐Tyr (nmol/L)	13.1 (7.9‐23.2)	11.9 (5.2‐21.6)	.543
Serum p‐Tyr/Phe (μmol/μmol)	0.8 (0.7‐1.1)	0.8 (0.6‐0.9)	.130
Serum m‐Tyr/Phe (nmol/μmol)	0.2 (0.1‐0.6)	0.3 (0.1‐0.6)	.434

Note

All data are expressed as median (IQR: 25‐75%).

Abbreviations: m‐Tyr, meta‐tyrosine; NSTEMI, non‐ST‐segment elevation myocardial infarction; o‐Tyr, ortho‐tyrosine; Phe, phenylalanine; p‐Tyr, para‐tyrosine.

As shown in Table 4, there were no significant differences between patients with STEMI and NSTEMI with regard to serum levels of Phe and Tyr isomers, whether distal to the culprit lesion or at the aortic root.

**TABLE 4:** Comparison of the amino acid parameters for patients with ACS

Parameters	Location	STEMI	NSTEMI	*P* value
Serum Phe (μmol/L)	Aortic root	35.5 (26.7‐44.9)	37.4 (34.0‐46.9)	.149
	The culprit lesion	44.7 (39.0‐58.6)	40.2 (33.6‐47.8)	.283
Serum p‐Tyr (μmol/L)	Aortic root	31.2 (26.7‐41.6)	35.6 (30.5‐40.6)	.353
	The culprit lesion	32.0 (30.2‐37.0)	32.8 (26.5‐40.2)	.630
Serum m‐Tyr (nmol/L)	Aortic root	17.6 (10.1‐36.2)	11.6 (6.7‐57.2)	.227
	The culprit lesion	10.39 (7.1‐37.1)	18.4 (6.1‐38.8)	.376
Serum o‐Tyr (nmol/L)	Aortic root	16.6 (6.6‐32.6)	13.1 (7.9‐23.2)	.597
	The culprit lesion	11.9 (6.9‐36.7)	11.9 (5.2‐21.6)	.488
Serum p‐Tyr/Phe (μmol/μmol)	Aortic root	0.9 (0.7‐1.1)	0.8 (0.7‐1.1)	.638
	The culprit lesion	0.7 (0.6‐0.8)	0.8 (0.6‐0.9)	.541
Serum m‐Tyr/Phe (nmol/μmol)	Aortic root	0.4 (0.2‐0.6)	0.2 (0.1‐0.6)	.200
	The culprit lesion	0.1 (0.1‐0.4)	0.3 (0.1‐0.6)	.366

Note

All data are expressed as median (IQR: 25‐75%).

Abbreviations: ACS, acute coronary syndrome; m‐Tyr, meta‐tyrosine; o‐Tyr, ortho‐tyrosine; Phe, phenylalanine; p‐Tyr, para‐tyrosine; STEMI, ST‐segment elevation myocardial infarction.

### 3.3 Correlation between serum amino acid parameters and baseline patient characteristics

**FIGURE 2:**
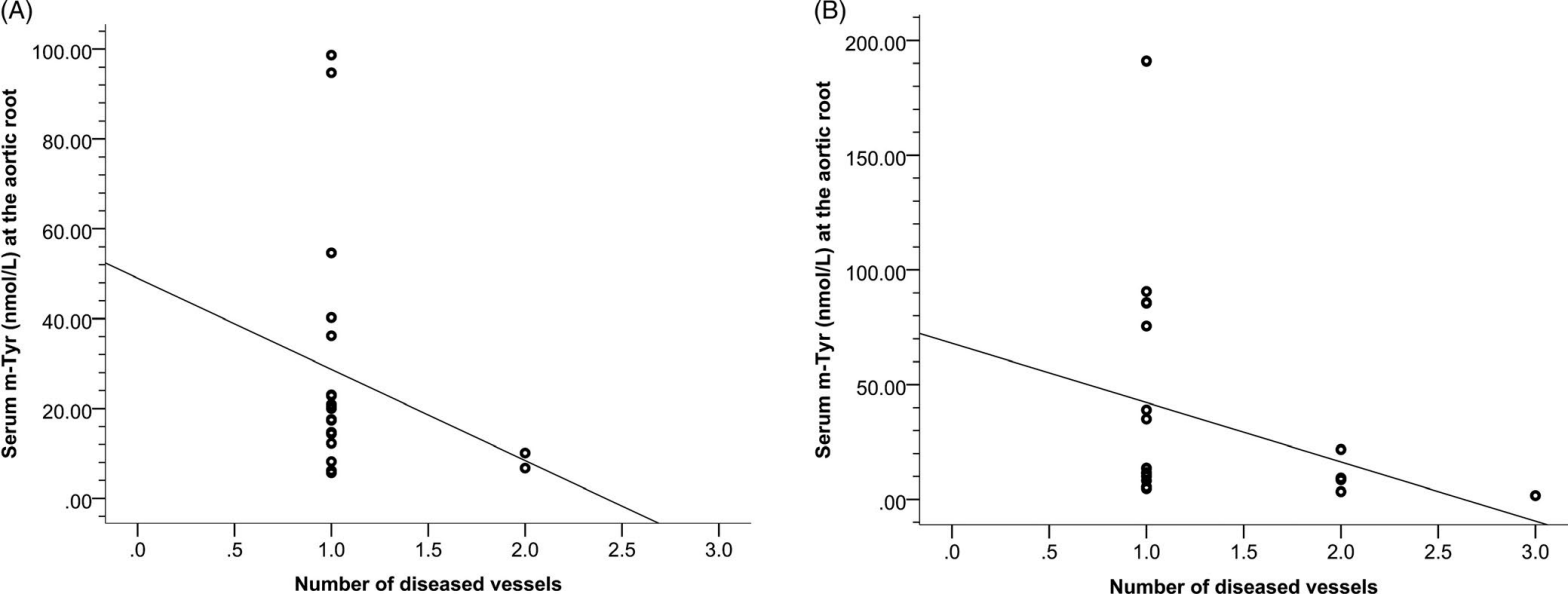
Scatter plot of serum m‐Tyr levels at the aortic root vs the extent of CAD (represented as number of diseased vessels) in patients with (A) STEMI and (B) NSTEMI

We examined the associations of the amino acid parameters with demographics and clinical data for patients, according to their diagnoses. Subject age, gender, smoking status, hypertension, and diabetes mellitus all failed to show any significant correlation with amino acid parameters at the aortic root or distal to the culprit lesion in patients with STEMI and NSTEMI (data not shown). Serum m‐Tyr levels at the aortic root showed a negative correlation with the extent of CAD in patients with NSTEMI (*ρ* = −0.446, *r* ^2^ = .096; *P* = .043), whereas there was no significant correlation in patients with STEMI (*ρ* = −0.236, *r* ^2^ = .050; *P* = .129) (Figure 2).

## 4 DISCUSSION

As mentioned in the literature, the pathological processes underlying vascular diseases are not fully understood; however, there is increasing evidence that oxidative stress and inflammation are positively associated with the rupture of atherosclerotic plaques and the incidence of ACS.^11^ The findings from this study suggest that certain oxidative stress markers may be associated with the extent of myocardial damage located proximally to the aortic root or distally from the culprit lesion in patients with STEMI and in those with NSTEMI.

In the current study, there were significantly higher levels of serum Phe distal from the culprit lesion compared to the aortic root in patients with STEMI; while there were slightly higher levels in patients with NSTEMI, this difference was not significant. Similar results have been described in patients suffering from diseases associated with inflammation and immune activation, such as ovarian carcinoma, HIV‐1 infection, and sepsis, as well as in patients after trauma and acute ischemic stroke.^25^, ^26^, ^27^, ^28^ These findings may be explained by the fact that increased serum Phe levels can be caused by the diminished conversion of Phe into Tyr by phenylalanine hydroxylase.^25^, ^26^, ^27^, ^28^, ^29^ The observed increase in serum Phe levels in STEMI patients could be attributed to the number of damaged cells and disruptions in tissue function.

In the present study, there was no evidence of a statistically significant difference between serum levels of p‐Tyr, m‐Tyr, and o‐Tyr distal to the culprit lesion compared to the aortic root in patients with STEMI and NSTEMI. Furthermore, serum concentrations of Phe and Tyr isomers did not show any significant differences between patients with STEMI and NSTEMI, whether distal to the culprit lesion or at the aortic root. A possible explanation for this finding may be the brief period of ischemia, as there is a short time period between the clinical data obtained and the duration of ischemia. Several reports have shown that elevations in some tyrosine isomers correspond to the duration of ischemia, indicating that hydroxyl radical production is associated with prolonged periods of ischemia.^14^, ^17^, ^30^ Another possible explanation is that only a small number of patients were studied, which may bias the results obtained during the study.

However, the results of the current study revealed that serum p‐Tyr levels were slightly higher, while serum m‐Tyr and o‐Tyr levels were slightly lower, in the distal region of the culprit vessel compared to the aortic root in patients with STEMI. In contrast, serum p‐Tyr and o‐Tyr levels were slightly lower, while serum m‐Tyr levels were slightly higher, in the distal region of the culprit vessel compared to the aortic root in patients with NSTEMI. These findings were unexpected and suggest that serum p‐Tyr levels clearly differ from those of m‐Tyr and o‐Tyr in patients with STEMI and NSTEMI. A possible cause of this difference may be the two pathways of tyrosine isomer synthesis: p‐Tyr is primarily produced enzymatically under physiological conditions, mainly in the kidneys, and is synthesized to a much lower extent under conditions of oxidative stress, whereas m‐Tyr and o‐Tyr are only formed nonenzymatically under conditions of oxidative stress.^17^

The results of this study showed no significant association between serum amino acid parameters and baseline patient characteristics except for serum m‐Tyr levels; they are negatively correlated with the extent of CAD at the aortic root in patients with NSTEMI. These results suggest that serum amino acid changes may be caused by the effects of oxidative stress and inflammation during myocardial infarction.

The clinical significance of this study is to discover that changes in the Phe and Tyr isomers (m‐, o‐, and p‐Tyr) are associated with oxidative stress after myocardial injury, which may play a role in chronic inflammation during initiation and progression of ACS. The limitation of this study is that only a small number of patients have been studied and a time period of ischemia is not specified. This research has thrown up many questions in need of further investigation.

## 5 CONCLUSIONS

Our data suggest that changes in serum levels of different Tyr isomers can mediate the effects of oxidative stress during myocardial infarction. The contribution of this study is to confirm the association of changes in the Phe and Tyr isomers with oxidative stress following myocardial injury.
